# 6D-ViCuT: Six degree-of-freedom visual cuboid tracking dataset for manual packing of cargo in warehouses

**DOI:** 10.1016/j.dib.2023.109385

**Published:** 2023-07-13

**Authors:** Guillermo A. Camacho-Muñoz, Juan Camilo Martínez Franco, Sandra Esperanza Nope-Rodríguez, Humberto Loaiza-Correa, Sebastián Gil-Parga, David Álvarez-Martínez

**Affiliations:** a Universidad del Valle, Cali, Colombia; b Universidad de los Andes, Bogotá, Colombia

**Keywords:** Intralogistics, Industrial metaverse, Packing of cargo, Point clouds, RGBD images, 6D pose estimation, Visual tracking, Visual cuboid tracking, Visual box tracking

## Abstract

Visual tracking of objects is a fundamental technology for industry 4.0, allowing the integration of digital content and real-world objects. The industrial operation known as manual cargo packing can benefit from the visual tracking of objects. No dataset exists to evaluate the visual tracking algorithms on manual packing scenarios. To close this gap, this article presents 6D-ViCuT, a dataset of images, and 6D pose ground truth of cuboids in a manual packing operation in intralogistics. The initial release of the dataset comprehends 28 sessions acquired in a space that rebuilds a manual packing zone: indoors, area of (6 × 4 × 2) m^3^, and warehouse illumination. The data acquisition experiment involves capturing images from fixed and mobile RGBD devices and a motion capture system while an operator performs a manual packing operation. Each session contains between 6 and 18 boxes from an available set of 10 types, with each type varying in height, width, depth, and texture. Each session had a duration in the range of 1 to 5 minutes. Each session exhibits operator speed and box type differences (box texture, size heterogeneity, occlusion).

Specifications table

**Table:** 

Subject	**Computer Science**, Human-Computer Interaction (HCI) **Engineering**, Industrial Engineering (IE), Intralogistics
Specific subject area	Visual tracking of objects from signals that belong to multiple sensors. Manual packing of cargo in the context of intralogistics operations.
Type of data	Images, Table, Figures
How the data were acquired	An operator grasps and transports each box from a consolidation to a packing zone. In the meanwhile, the session is scanned by three devices: 1. A Microsoft HoloLens-2 (HL2) is attached to the operator's head and allows the acquisition of RGBD images and camera 6D pose in space. 2. A Microsoft Kinect v2 was fixed with a line of sight to the consolidation zone to acquire RGB-D images. 3. A Phase Space motion capture (MoCap) system was set up to cover the workspace. Two active markers were attached to each box's top side. Five additional markers were attached to the HL2 device.
Data format	Raw: • Color images from the mobile sensor (760 × 428 px resolution), PNG format • Depth Images from the mobile sensor (320 × 288 px resolution), PGM format • HL2 device poses for each keyframe and from the on-device tracker, TXT format. • Color images from the fixed sensor (1920 × 1080 px resolution), MP4 format • Depth images from the fixed sensor (512 × 424 px resolution), NPZ format • 3D position of MoCap markers in millimeters, C3D format Analyzed • Matrix transformations between HL2 coordinate frame and MoCap coordinate frame, TXT format. • Target box at each HL2 frame, TXT format Filtered • Point Clouds from mobile sensor signals, PLY format. • 6D pose of boxes from MoCap signals, CSV format. This data is available for initial instants and relocation instants. • Session descriptors (number of boxes, occlusion level, operator's speed level, and 6D initial pose of boxes from MoCap signals), CSV format Miscellaneous • Box descriptors (length, depth, width, type, texture), CSV format • Intrinsic parameters of depth and color Kinect cameras, CSV format
Description of data collection	Data were acquired indoors in an area isolated from sunlight and with tube light illumination. The workspace rebuilds a dispatching zone from a warehouse. The operator performs a manual packing operation while sensors are scanning the scene. Each session was defined to obtain a different combination of four factors: occlusion, the texture of the cargo, cargo size heterogeneity, and speed of the operator. Each of the first three factors has three levels, and the last factor has two levels.
Data source location	Institution: Universidad de los Andes, Engineering Department, Colivri Laboratory. City/Town/Region: Bogotá, Colombia. Latitude and Longitude: 4° 36′ 2.53 “N, -74° 03′ 33.83” W.
Data accessibility	Repository name: Dryad Data identification number: https://doi.org/10.5061/dryad.jq2bvq8dv Direct URL to data: https://datadryad.org/stash/share/EE0sEaiydea9XLlTy1lXY-SMDmRAWef-IppfoYwQAYM

## Value of the Data

• 6D-ViCuT dataset explores the manual packing of cargo configuration, which defines new challenges and opportunities when approaching the 6D visual tracking system to support the packing operation in logistic fields.
• This dataset can be used to evaluate 6D object pose estimation and tracking algorithms from RGB images, depth images, point clouds, and combinations of previous images from single and mobile cameras. Furthermore, the dataset can be used to compute accurate performance metrics of estimated poses for cuboids under realistic packing conditions based on (1) the 6D pose ground truth provided by the MoCap system and (2) the provided transformation matrix between the capture systems
• The outside-in arrangement uses fixed cameras to scan the cargo and the operator. This arrangement allows the assessment of 6D visual tracking algorithms in automatic support systems for packing with industrial robots and collaborative robots.
• The inside-out arrangement uses mobile cameras mounted on the head of the operator under controllable conditions of camera speed. This arrangement is helpful when developing applications supporting the operator in the 6D detection of cargo and the guidance to assemble the optimal packing pattern using augmented reality technology. Consequently, this dataset helps develop computer vision algorithms that support the industry of the metaverse.
• The moving of boxes during the packing operation and the variability in selected factors of each session allow testing algorithms in challenging conditions of dynamic environments, blur associated with camera shaking, occlusion between cuboids, different levels of texture on the surface of the cuboid, and heterogeneity in cuboid size, between others.
• The image dataset can be used to adapt pre-trained models for object pose estimation to work on cuboids under packing conditions. Furthermore, manipulating the existing images and annotations in the dataset makes it possible to create new training data to improve the performance of an algorithm, especially in data-driven approaches.

## 1 Objective

The objective behind the generation of this dataset was to create a dataset to evaluate pose estimation and visual tracking algorithms under fixed and mobile camera approaches and with realistic manual packing scenarios: different levels of texture in the cuboids, the inclusion of multiple cuboids in the scene with stacked and non-stacked layouts, different levels of heterogeneity in the size of the boxes and accurate ground-truth for the pose of the boxes.

## 2 Data Description

**Fig. 1:**
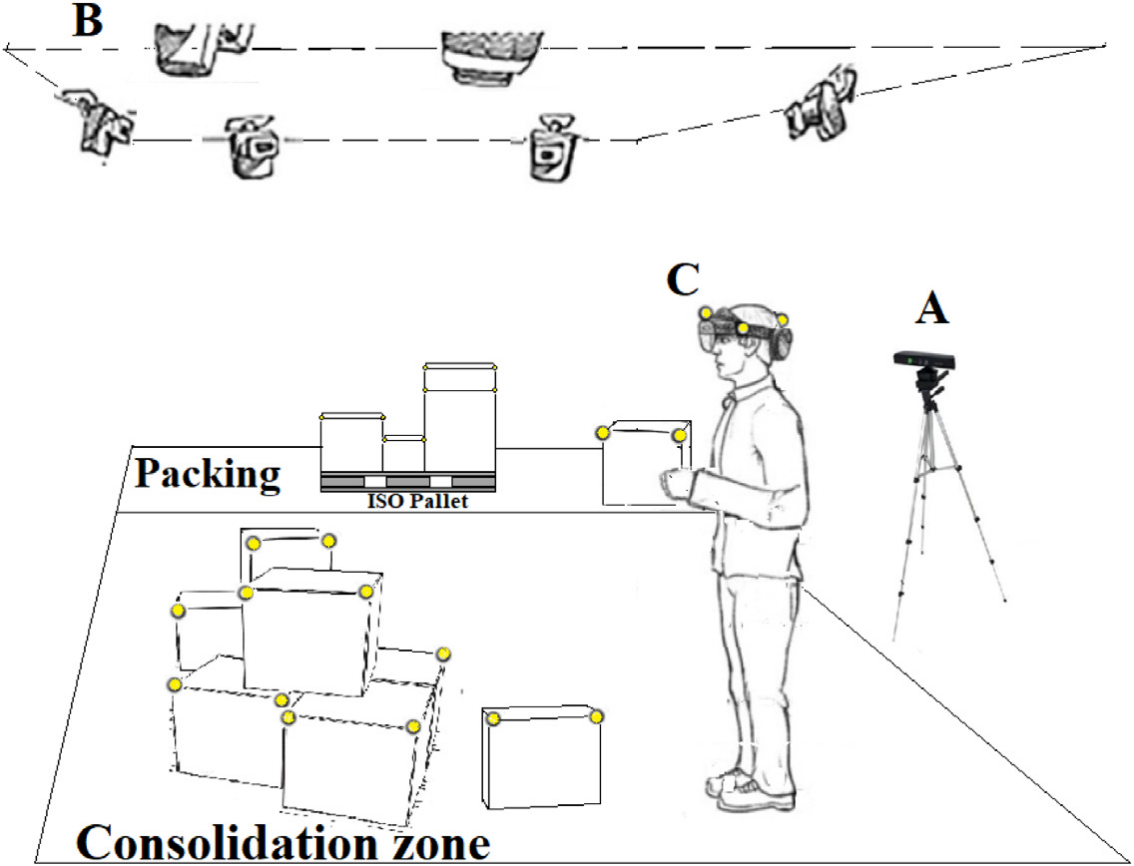
Schematic of the workspace where the data were acquired with operator, cuboids, pallet and instruments. A: fixed RGBD camera. B: motion capture system installed on and structure located at 3.36 m of the floor and composed of 12 cameras and active markers represented as yellow circles. C: HoloLens 2 device. Figure adapted from [6].

6D ViCuT dataset is directly focused on cargo packing operations in distribution centers, aiming to maximize space utilization, subject to practical constraints. Packing of cargo can be described as a geometrical assignation problem between the cargo and the container, where the solution is a **packing pattern** that assigns to each cargo a 6D pose in the container. Although some algorithms compute the optimal solution, it is challenging for an operator to interpret the instructions and assemble the optimal pattern. The development of automatic or operator support systems for assembling the **optimal packing pattern** requires the 6D detection of the cargo, which, in addition to being heterogeneous in its size and texture, usually rests in a consolidation zone in stacked configurations within cluttered scenes and high occlusion. These features are challenging to develop the 6D visual tracking systems for objects in packing applications. The evolution of trackers in the logistics field has been founded in datasets such as BigBird [1], APC [2], ARMBench [3], and recent Amazon dataset used in [4]. These datasets explore a standard configuration where the cargo resides in a small container [1,2,3] or conveyor [4] reachable by an industrial manipulator. None of these datasets explores the configuration of a mobile operator, simultaneously solving the exploration of a consolidation area in search of the cargo, the manual picking of the cargo, and the manual accommodation of cargo in the container [5]. This configuration, which we have called “**manual packing of cargo**,” has not been widely addressed by the community specialized in visual tracking algorithms, even though it is a frequent configuration in warehouses. The 6D tracking dataset provided in this work differs from other datasets in logistics because it has been designed to explore the manual packing of cargo configuration, with controlled combinations of cargo heterogeneity, cargo occlusion, and cargo texture, until three levels of stacking in sessions and two spatial sensor arrangements: outside-in and inside-out. The dataset was acquired indoors in an area isolated from sunlight and with warehouse illumination based on tube lights. In the working area (6.8 × 4.5 × 2 m^3^), the consolidation and packing zones were defined as illustrated in Fig. 1, recreating the packing process of a leading package transport company in the region. The objects to be packed are cuboids located in the consolidation zone. The distribution of cuboids in this area does not follow a predefined pattern and can present stacked or non-stacked distributions. The packing zone contains an ISO pallet (0.9 × 1.2 m^2^) where the operator will deposit all the cuboids from the consolidation zone in a predefined pattern: **optimal packing pattern**. This pattern is computed by an application that uses descriptors of boxes in **the consolidation zone** and the descriptors of the pallet as inputs. The application also computes the physical packing sequence (PPS), which describes the order in which each cuboid enters the pallet or, seen in another way, the pick-up sequence of cuboids in the consolidation zone. The consolidation zone has an approximated area of (4.7 × 4.5 m^2^) and the packing zone has an approximated area of (2.1 × 4.5 m^2^).

**Fig. 2:**
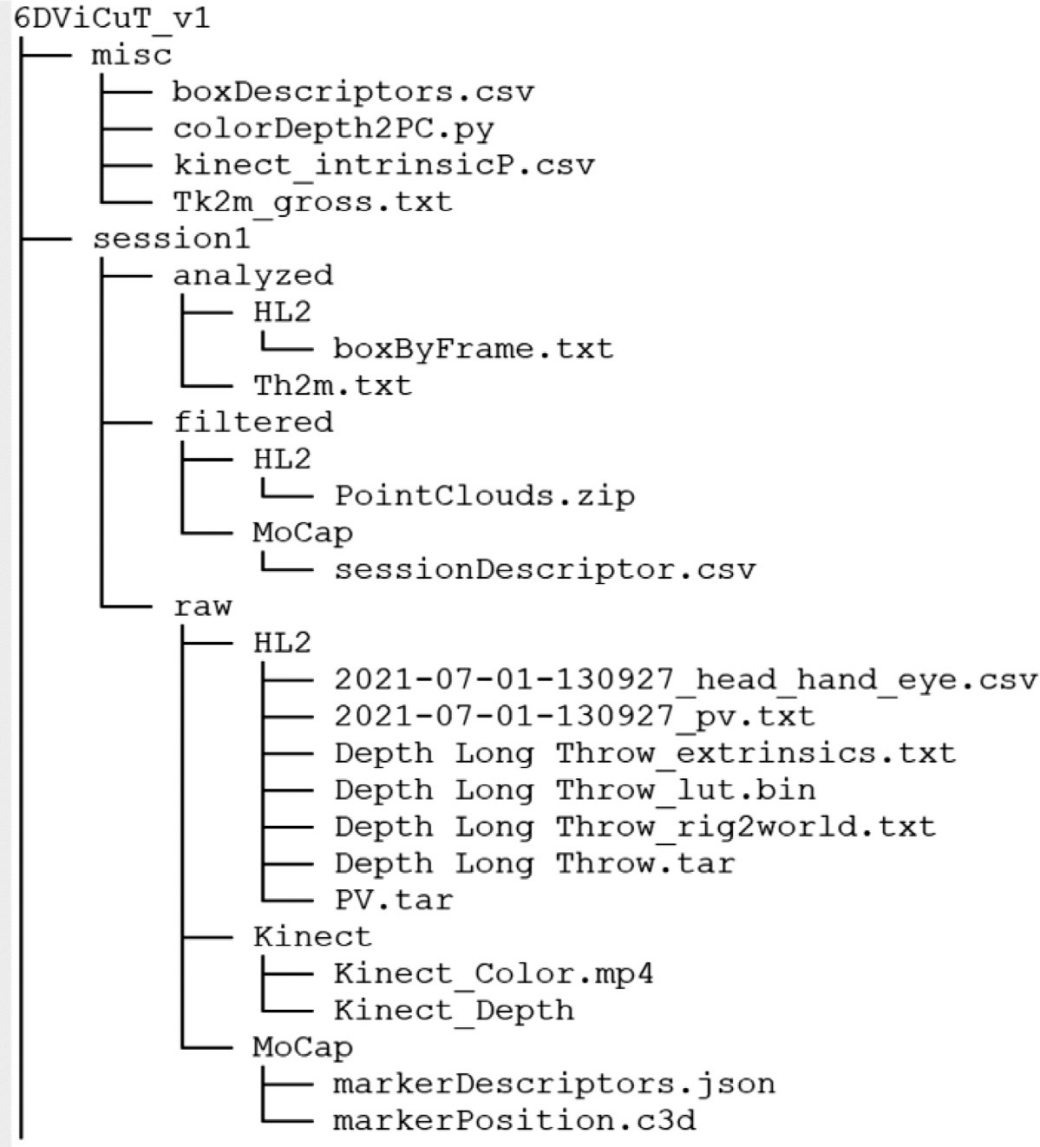
Sample of tree diagram of folders and files in the dataset. The sample is limited to folder misc and session1. Data associated to the other sessions keeps the same structure.

The dataset comprehends 28 sessions. A session is a process where simultaneously the manual packing of all the cuboids in the consolidation zone and the scanning of the workspace with the available devices are performed. A single operator with a height of 1.65 m performed all the manual packing sessions. Data generated at each session is available in a folder named session***x***, where ***x*** represents the session's identifier. Fig. 2 presents a sample of a tree diagram with the structure of folders and files in the dataset, for session 1 and misc folder. In the following paragraphs, this struct will be described in detail.

**Notation**. $T_{i}^{j}\in SE\left(3\right)$is denoted as a 6D rigid transformation encompassing rotation and translation, that transforms a point in coordinate frame $q_{i}$ to $q_{j}$. $T_{i}^{j}$ is presented in Equation (1)

(1) $$T_{i}^{j}=\left[\begin{array}{cccc} r_{11} & r_{12} & r_{13} & p_{x}\\ r_{21} & r_{22} & r_{23} & p_{y}\\ r_{31} & r_{32} & r_{33} & p_{z}\\ 0 & 0 & 0 & 1 \end{array}\right]$$

$q_{m}$ corresponds with MoCap coordinate frame.

$q_{h}$ corresponds with world in HL2 device coordinate frame.

$q_{c_{h}}$ corresponds with camera rig coordinate frame in HL2 device.

$q_{boxID}$ corresponds with the box coordinate frame. This one is attached to the geometric center of the upper face of a box.

$q_{k}$ corresponds with Kinect device coordinate frame.

The experiment used 30 cardboard boxes as cuboids with properties available in the folder misc with the name **boxDescriptors.csv**; a detailed description of those boxes is presented in section materials.

### 2.1 Kinect v2

**Fig. 3:**
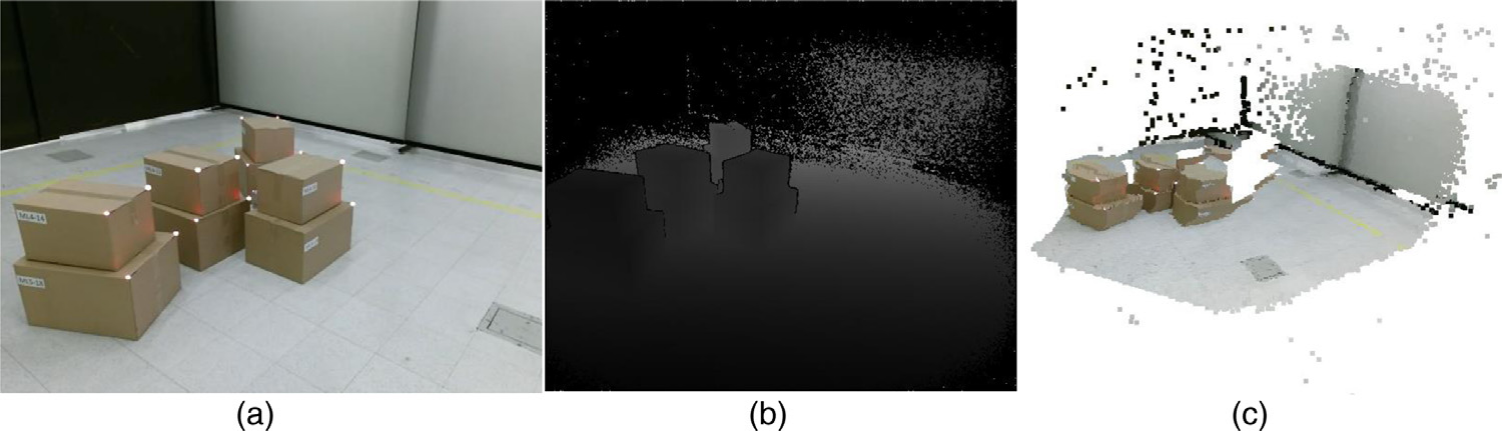
Sample of the images acquired with the Kinect Device: boxes in the consolidation zone. (a) 1920 × 1080 px color image, (b) 512 × 424 px depth image, and (3) colored point cloud (up to 217088 3D points).

A Kinect v2 was located with the sight of view to the consolidation zone (height=1.3 m), as depicted in Fig. 1. The raw data from this sensor is in the path /raw/Kinect/ and is composed of color images (**Kinect_Color.mp4**) and depth images (**Kinect_Depth.npz**). The intrinsic parameters of color and depth cameras (**kinect_intrinsicP.csv**) and a script (**colorDept2PC.py**) to obtain point clouds from color and depth images are shared in the misc folder. Fig. 3 presents a sample of the acquired images with this sensor and a generated point cloud.

The transformation matrix $T_{k}^{m}$ to project the Kinect coordinate frame ($q_{k}$) to the MoCap coordinate frame ($q_{m}$) is available in misc folder with the name Tk2m.txt. This matrix is a gross approximation computed from the 3D positions of markers attached to the Kinect during the enlistment of the experiment. This matrix can be refined with a procedure similar to the one described in the methods section called “Computing the projection matrix $T_{h}^{m}$”.

### 2.2 Motion capture system (MoCap)

**Fig. 4:**
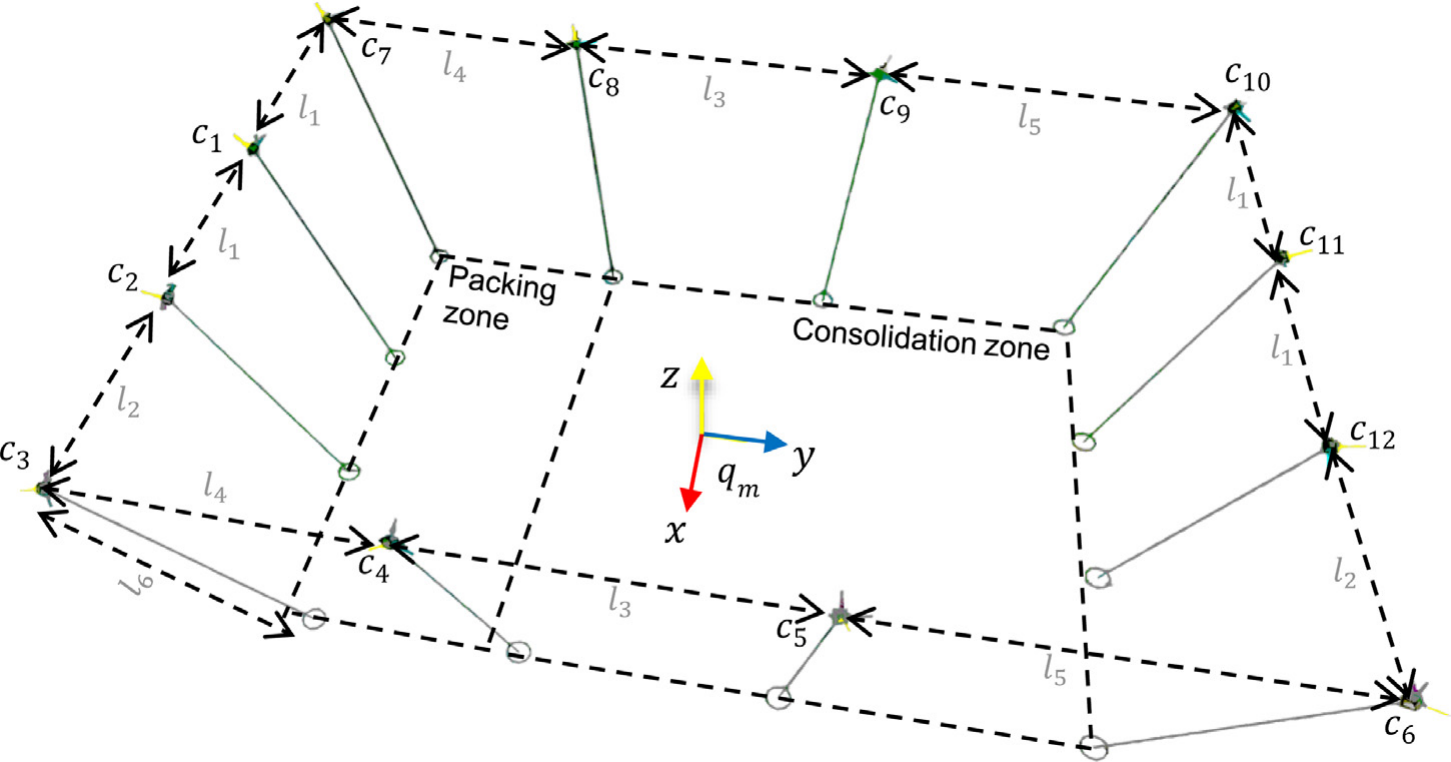
Distribution of twelve MoCap cameras $c_{x}$ in the workspace with x=1..12. The coordinate frame in the figure corresponds with the MoCap coordinate frame.

Twelve cameras from the MoCap System were placed around the workspace in a rectangular arrangement, using as support a structure located at 3.38 m of the floor, as illustrated in Fig. 4; ($l_{1}=1.45\;m,\;l_{2}=1.6\;m,\;l_{3}=2.05\;m,\;l_{4}=2.1\;m,\;l_{5}=2.65\;m,$ and $l_{6}=3.38\;m$). This Figure also illustrates the localization of the MoCap coordinate frame $q_{m}$ system in the workspace. In order to accurately capture markers in the whole workspace, cameras were positioned as indicated by the vendor: with some overlap in their field of view and a few meters or more apart. The raw data from this device is available at path raw/MoCap and is composed of files **markerPosition.c3d** and **markerPosition.json**. The first file contains the information needed to read the 3D positions of each marker scanned in the session in standard format C3D [7], recorded in millimeters. The second file contains metadata about the scanned session: date, frames, frequency, and objects associated with each marker ID, among others.

**Fig. 5:**
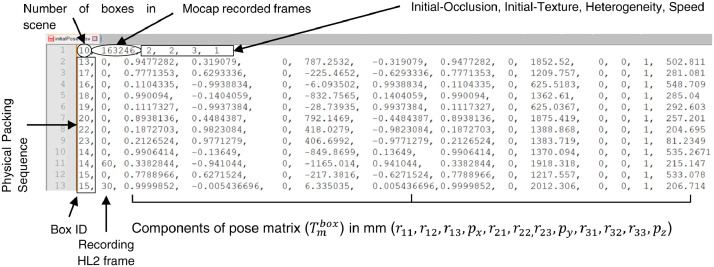
Components of the file sessionDescriptor.csv for session 5. A description of factors is presented in the section Experimental Design.

Following a strict picking sequence of boxes (physical packing sequence - PPS) in the consolidation zone is necessary to optimize the cost function associated with the packing operation. During this picking and in sessions with stacked boxes, in some cases, it was necessary to move a box from a superior level to reach another box in a lower level, giving rise to what we call relocation of a box. MoCap raw data was used to compute the 6D pose for the box's initial and relocation times. The procedure is presented in the methods section. The result of this procedure is combined with session descriptors in a file located at /filtered/mocap/ with the name **sessionDescriptors.csv**. Fig. 5 presents a sample of the sessionDescriptors.csv file for session 5. The first row contains the descriptors of the session; in order of appearance, they are:

• The number of boxes for the example in Fig. 5 is 10.
• The number of MoCap recorded frames with a value of 163246 for session five.
• The level of each factor in the sequence: initial-occlusion, initial-texture, heterogeneity, and speed, with values of 2, 2, 3, 1, respectively, for the example. In the Experimental Design section is the correspondence between those values and the levels of each factor.
• Each of the following rows contains information about the box pose in order of appearance:
  ○ **Box identifier (ID)**. It is a unique identifier corresponding to the last number on the label attached to the box, as illustrated in Fig. 10. In principle, **sessionDescriptors** file has as many IDs as boxes used in the session. However, when the process requires relocating boxes, one or more IDs will repeat, as illustrated in rows 10 to 13 of Fig. 10. Additionally, as depicted in Fig. 10, the file presents the box IDs in a sequence corresponding to the physical packing sequence used in the session.
  ○ **Recording HL2 frame.** Frame at which the pose was measured concerning the HL2 sampling system. When this value is zero, it indicates the initial frame. When this value is different from zero, it indicates the frame at which relocation occurs. This datum allows identifying the frames at which MoCap poses must be updated when assessing pose estimation from images that were acquired from HL2 device.
  ○ **Components of the pose matrix**. Correspond in appearance order to $r_{11},r_{12},r_{13},p_{x},r_{21},r_{22},r_{23},p_{y},r_{31},r_{32},r_{33},p_{z}$, taking as reference Equation 1. This transformation matrix allows moving the MoCap coordinate reference system $q_{m}$ to a coordinate reference system located in the geometric center of the upper face of each box $q_{boxID}$. The methods section explains the procedure to obtain these values.

### 2.3 HoloLens-2

A HoloLens-2 (HL2) was mounted to the operator's head during each session. Therefore, considering the operator's height, the HL2s were at a maximum distance of 1.65 m from the ground. The data from this device use timestamp-based synchronization. Timestamp is an integer value representing the quantity of 100 ns intervals that have passed since January 1, 1601 (UTC), until a file was created. The raw data is available in the path /raw/HL2/. The folder contains three main files which will be described below; the rest of files are required to unzip the .tar files as described in [8] in the section “Python postprocessing”.

**Fig. 6:**
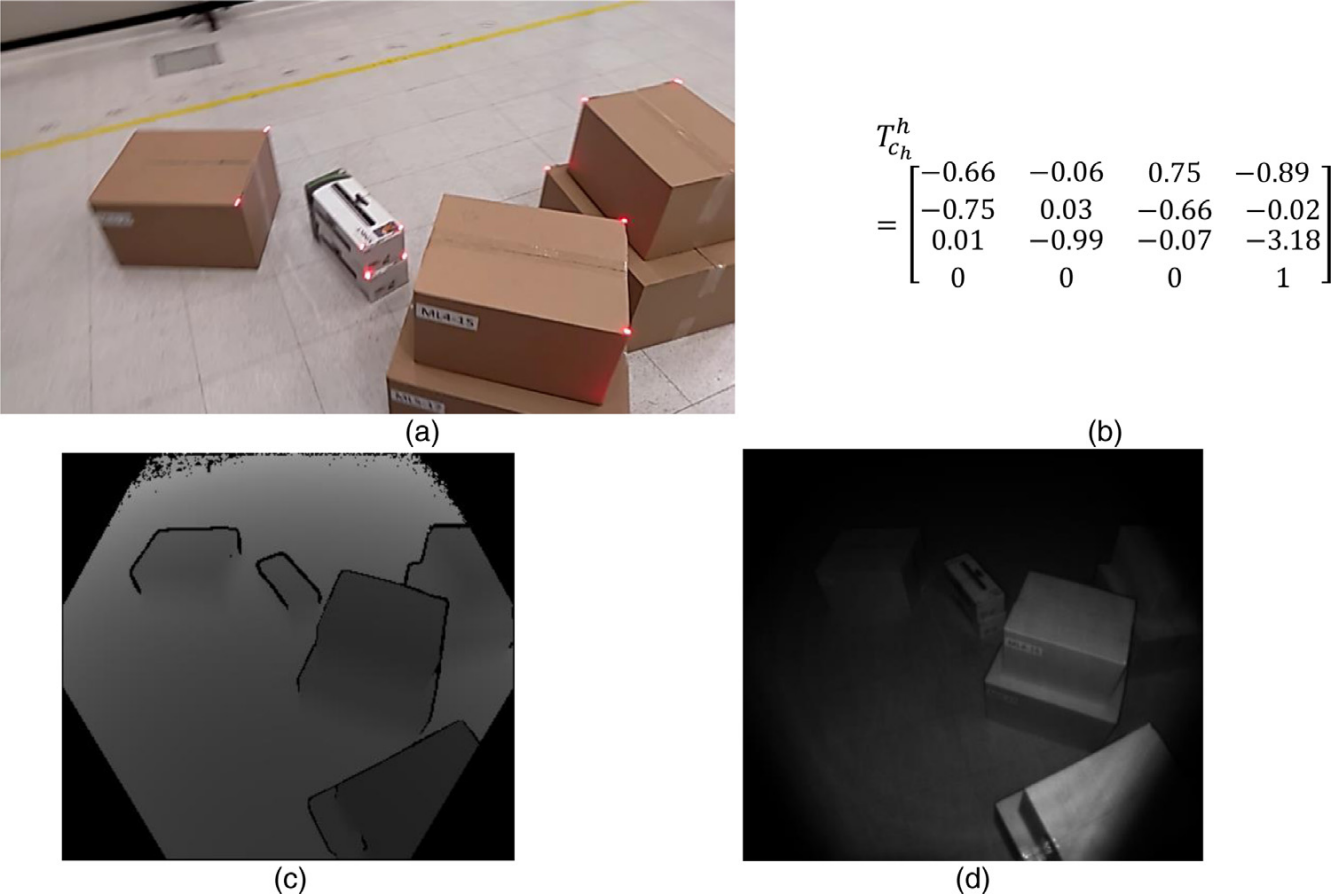
Samples of data acquired with the HL2 sensor in one of the frames of session 5. (a) RGB image with name 132697151634846737.png, (b) rig coordinate frame to HL2-world transformation matrix $T_{c_{h}}^{h}$ with timestamp 132697151634073057, (c) Depth Long Throw image with name 132697151634073057.pgm, (d) Depth Long Throw with Active Brightness and name 132697151634073057_ab.pgm.

1. The **PV.tar** file contains the color images compressed in PNG format. The name of each image file corresponds with the timestamp filetime. A sample of a color image for a single frame is presented in Fig. 6a. Note the presence of some blur in the image as a consequence of the camera's movement.
2. The “**Depth Long Throw.tar**” file contains compressed depth images in PGM format. The name of each image file corresponds with the timestamp filetime. A sample of the depth image for a single frame is presented with two versions in Fig. 6: depth measured in meters (Fig. 6c) and depth with amplitude of the return laser signal or active brightness (Fig. 6.d).
3. The “**DepthLongThrow_rig2world.txt**” file contains one row per depth frame, with (1) timestamp and (2) the components of the transformation matrix $T_{c_{h}}^{h}$ in the sequence . $T_{c_{h}}^{h}$ allows moving the coordinate frame $q_{c_{h}}$ to the coordinate frame $q_{h}$, as depicted in Fig. 8. The coordinate frame $q_{c_{h}}$ is attached to the camera rig of the HL2 device, then changes with each device movement during the session. The coordinate frame $q_{h}$ is the world coordinate frame for HL2 device, which is kept fixed during the session; $q_{h}$ is selected as the HL2 device pose at the beginning of the session. Fig. 6b presents a sample of numeric values for the matrix $T_{h}^{c_{h}}$ in a single frame; the distance is recorded in meters.

**Fig. 7:**
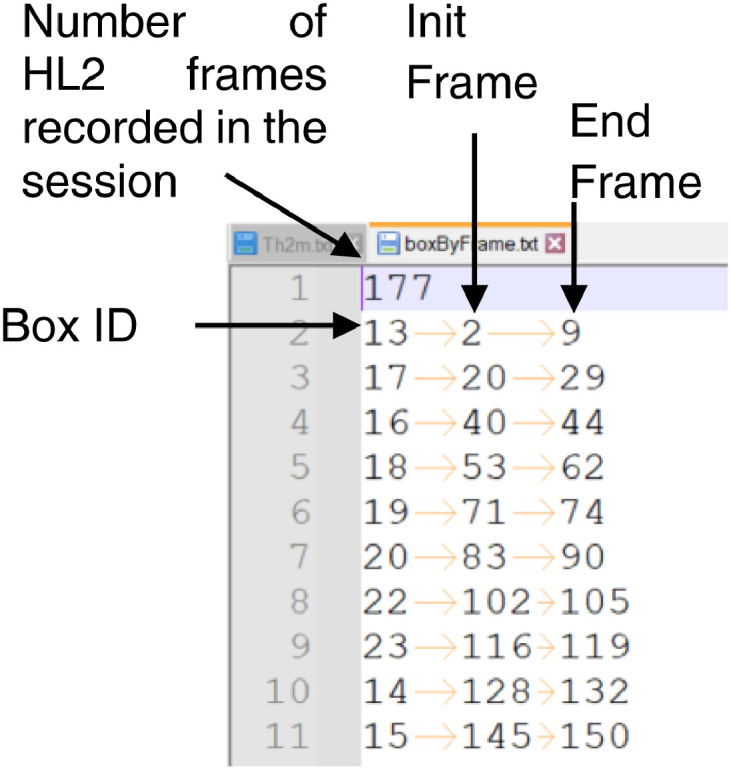
Format of boxByFrame.txt file.

During each session, the operator is instructed to follow a physical packing sequence (PPS) which defines a target box each time he enters the consolidation zone. In the file boxByFrame.txt, the target box for each HL2 frame at which the operator was seeking a box is recorded. This file is available in the path analyzed/HL2. Fig. 7 presents a sample of this file where the information is sorted as follows: the first row contains a number corresponding to the recorded HL2 frames during the session, 177 for the example. Each of the following rows contains three data: (1) the target box ID, (2) the frame at which the operator began the seek, and (3) the frame at which the operator reached the target box; for the example in row two of Fig. 7, those values are 13, 2, and 9, respectively. It is assumed that the range of frames between the last number of a row and the first frame in the next row was used by the operator to transport the target box to the packing zone, place the box on the pallet and recover the visibility of the consolidation zone, e.g., frames 10 to 19 between rows two and three of Fig. 7.

**Fig. 8:**
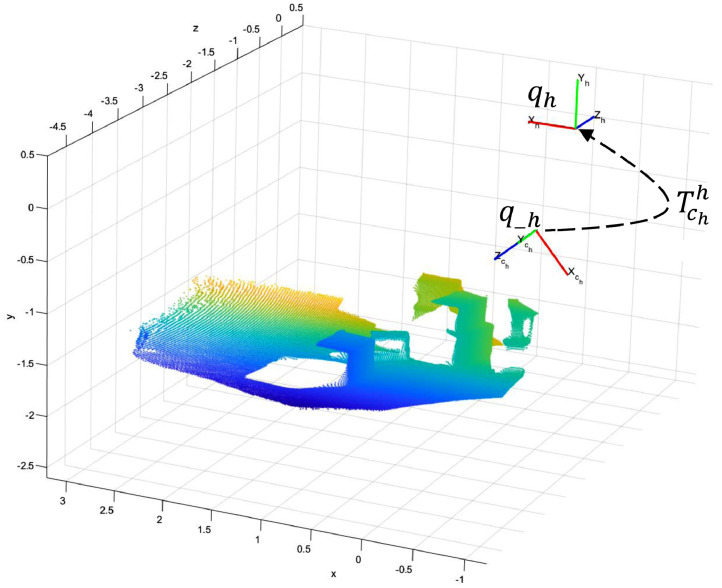
Non-colored point cloud generated from raw HL2 data in frame 25 of session 5, coordinate frames $q_{h}$, $q_{c_{h}}$, and rigid transformation matrix $T_{c_{h}}^{h}$. Frame 25 corresponds with the timestamp 132697151634073057.

Non-colored point clouds were computed from raw HL2’s RGB-D images. Those files are available in the path /filtered/HL2/PointClouds, and the procedure to compute this data is presented in section methods. This computation was performed for frames where the operator searches a target box. The points in those images are associated with the box, the floor, and the background, as depicted in Fig. 8, which is referenced to the $q_{h}$ coordinate system. Therefore, the color in Fig. 8 represents the distance between a point and the origin of $q_{h}$.

### 2.4 Transformation between MoCap and HL2 coordinate frames

**Fig. 9:**
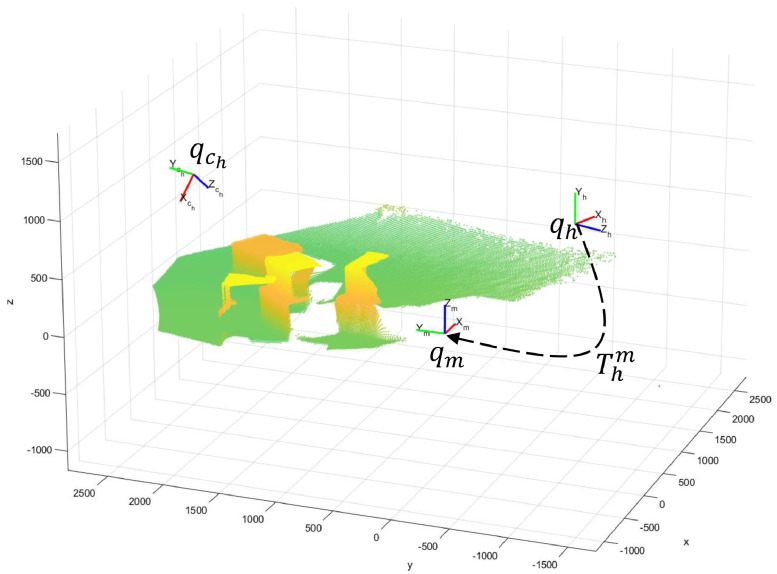
The point cloud of frame 25, session 5, projected to the $q_{m}$ coordinate frame.

From HL2 raw data and MoCap filtered data (sessionDescriptors.csv), a projection matrix $T_{h}^{m}$ was computed between the coordinate frames $q_{h}$ and $q_{m}$. This file is named Th2m.txt and is available in the subfolder analyzed for each session. The matrix $T_{h}^{m}$ allows the project of a point from $q_{h}$ to $q_{m}$ and with this enables the direct comparison between analytics computed from (1) raw HL2 images and (2) measurements acquired with the MoCap system, which is used as a ground-truth in the 6D-ViCuT dataset. The procedure to obtain the $T_{h}^{m}$ matrix is presented in methods section. Fig. 9 presents the $T_{h}^{m}$ projection of the point cloud associated with frame 25, session 5.

## 3 Experimental Design, Materials and Methods

### 3.1 Experimental design

Since 6D-ViCuT dataset was created to evaluate realistic packing scenarios, 28 sessions were created with different combinations of four factors: initial occlusion, initial texture, heterogeneity of cuboid size, and operator speed. Table 1 reviews the variety of levels for each session. Our principal interest was in the non-stacked sessions; as a consequence, most sessions with low initial occlusion exist, this is 18 of 28 sessions.

**Table 1:** Level of factors for each of the 28 sessions in the dataset. $F_{1}$: Initial Occlusion, $F_{2}$: Initial Texture, $F_{3}$: Heterogeneity of box's size, $F_{4}$: Operator Speed. Levels for factors $F_{1}$ to $F_{3}$ are codified as (1) for Low, (2) for Medium, and (3) for High. For factor $F_{4}$, the codification is (1) for Low and (2) for High.

Session ID	$F_{1}$	$F_{2}$	$F_{3}$	$F_{4}$	Session ID	$F_{1}$	$F_{2}$	$F_{3}$	$F_{4}$
1	2	2	1	2	19	1	1	1	1
2	2	3	1	2	20	1	2	2	2
3	1	1	2	1	21	3	2	2	1
4	2	3	3	1	25	1	1	3	1
5	2	2	1	1	27	1	3	1	1
6	2	2	2	1	32	1	3	2	1
7	2	3	2	1	33	1	1	1	2
10	1	1	3	2	35	1	2	2	1
12	1	3	1	2	36	1	2	1	1
13	1	3	2	2	39	1	2	3	2
15	3	3	1	2	45	1	1	2	2
16	3	3	3	2	52	1	3	3	2
17	1	2	1	2	53	1	2	3	1
18	3	1	2	1	54	1	3	3	1

**Initial Occlusion (**$F_{1}$**).** Occlusion occurs when the projection of two or more objects overlaps and seemingly merges or combines in the image. This dataset uses the initial-occlusion factor, which defines the occlusion level of a session at the initial time, e.g., before the first box is grasped. This factor is categorized into three levels: 3 for high, 2 for medium, and 1 for low. The Initial occlusion factor is calculated as presented in Eqn 2

(2) $$\begin{array}{c} F_{1}=\left\{ \begin{array}{cc} \begin{array}{c} 3\\ 2\\ 1 \end{array} & \begin{array}{c} 0.4<o_{P}\;\left(0\right)<0.5\;\wedge \;\;N_{S}\;\left(0\right)=3\\ 0.2<o_{P}\;\left(0\right)<0.4\;\wedge \;\;N_{S}\;\left(0\right)=2\\ o_{P}\;\left(0\right)<0.1\;\wedge \;N_{s}\;\left(0\right)=0 \end{array} \end{array}\right. \end{array}$$

Where $o_{P}\left(0\right)$ is the occlusion level at the initial time and w.r.t. a fixed point of view P and $N_{s}\left(0\right)$ is the maximum number of boxes in a stack within the scene at the initial time. To compute $o_{P}\left(0\right)$, the 3D model of the consolidation zone (with boxes) is first rendered under two conditions: (1) considering individual boxes as individual objects, (2) considering all the boxes as a single object. The first condition implies an iterative process over all the boxes with three steps: render the individual box, count the number of pixels that belong to the box, and accumulate the number of pixels $p_{wo}$. In the second condition, the render is performed considering all the boxes, then pixels that belong to boxes are counted as $p_{o}$. Finally, the relationship $o_{P}\left(0\right)=\;\frac{p_{o}}{p_{wo}}$ is computed.

**Initial Texture (**$F_{2}$**)**. The texture is a property that refers to a spatially repeating pattern on an object's surface that can be sensed visually. As the materials section explains, all the used boxes present texture in high and low levels. Initial texture has been defined as a function of the number of boxes with high texture in the scene at the initial time. This factor is computed as follows,

(3) $$\begin{array}{c} F_{2}=\left\{ \begin{array}{c} 3\\ 2\\ 1 \end{array}\right.\begin{array}{cc} & \mathrm{Most}\;\mathrm{than}\;29\%\;\mathrm{of}\;\mathrm{the}\;\mathrm{boxes}\;\mathrm{present}\;\mathrm{high}\;\mathrm{textures}\\ & \;\;\mathrm{Between}\;\left[11-29\right]\;\%\;\mathrm{of}\;\mathrm{the}\;\mathrm{boxes}\;\mathrm{present}\;\mathrm{high}\;\mathrm{textures}\\ & \mathrm{None}\;\mathrm{of}\;\mathrm{the}\;\mathrm{boxes}\;\mathrm{present}\;\mathrm{high}\;\mathrm{textures}\;\mathrm{on}\;\mathrm{their}\;\mathrm{surface} \end{array} \end{array}$$

**Heterogeneity of box size (**$F_{3}$**)**. This factor describes the assortment of boxes in the consolidation zone at the initial time. It is computed in terms of box types and number of instances as follows,

(4) $$\begin{array}{c} F_{3}=\left\{ \begin{array}{c} 3\\ 2\\ 1 \end{array}\right.\begin{array}{cc} & \mathrm{Number}\;\mathrm{of}\;\mathrm{types}\;\geq 8\;\mathrm{and}\;\max\left(\mathrm{Number}\;\mathrm{of}\;\mathrm{instances}\;\mathrm{by}\;\mathrm{type}\right)=1\\ & \mathrm{There}\;\mathrm{exist}\;\mathrm{two}\;\mathrm{instances}\;\mathrm{of}\;\mathrm{each}\;\mathrm{box}\;\mathrm{type}\;\mathrm{in}\;\mathrm{the}\;\mathrm{scene}\\ & \max\left(\mathrm{Number}\;\mathrm{of}\;\mathrm{instances}\;\mathrm{by}\;\mathrm{type}\right)\geq 3 \end{array} \end{array}$$

**Operator speed (**$F_{4}$**)**. To control the operator speed, we used sounds during the session with two beats: (1) 122 bpm for low speed, (2) 212 bpm for high speed. The operator was instructed to adjust his velocity for those sounds.

### 3.2 Materials

**Illumination**. The workspace was illuminated with tube lights in a basement semi-isolated from daylight. The illuminance level of the scene was uncontrolled. The sessions were acquired in six working days. Working hours were between 8.00 am and 5.00 pm. The mean illuminance level in the morning was measured as 495 lux with a standard deviation of 15.18 lux. The afternoon's mean illuminance level was 385 lux with a standard deviation of 7.38 lux.

**Fig. 10:**
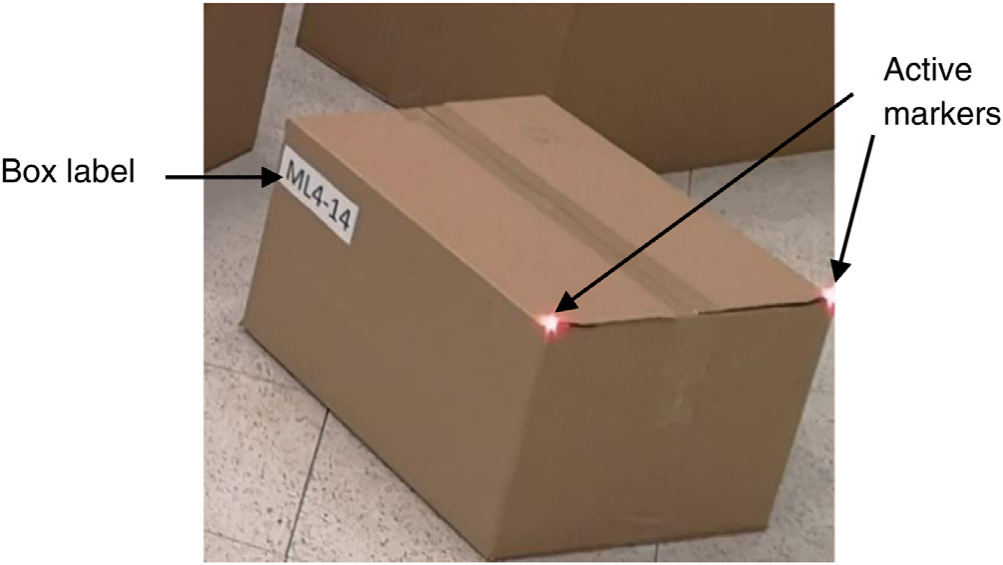
Sample of a standard size, low texture box used during the dataset acquisition. The image shows a label on the box and the position of the active markers on the box.

**Fig. 11:**
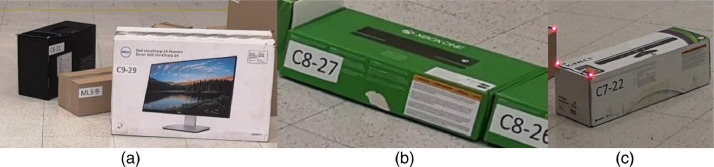
Sample of non-standard, high-texture boxes used during the dataset acquisition. (a) Box 21 is an example of type 6, box 29 is an example of type 9, (b) Box 27 is an example of type 8, and (c) Box 22 is an example of type 7.

**Cuboids**. For cuboids, we have used 30 cardboard boxes that belong to 10 predefined types, as reviewed in Table 2. The box size was selected taking as reference an actual packing operation where there exist two alternatives in box size selection: (1) one of the standard size boxes of the logistic operator (codified as ML), (2) a non-standard size box (codified as C). The second alternative is when the product has an acceptable box to transport the cargo, i.e., the original box. The number of instances of each type was selected from the desired heterogeneity of box size in Table 1. Each box was labeled with an identifier composed of a letter(s) followed by a number, a hyphen, and a number, e.g., ML4-14. The letters indicate a standard size (ML) or a non-standard size (C). The number that follows the letter(s) does not have a relevant meaning for the dataset. The number after the hyphen is the box ID, a unique identifier for the box. For example, the box in Fig. 10 is labeled “ML4-14”, which stands for standard size, and box ID 14. The box texture was selected to obtain 70% of the boxes with low and 30% with high textures. A low-texture box is a non-laminated cardboard like the one in Fig. 10. A high texture box is a laminated cardboard box with advertising logos printed on the surface. Samples of high-texture boxes are presented in Fig. 11.

**Table 2:** Box Types used during the data acquisition.

Type	Height (m)	Width (m)	Depth (m)	Number of instances	IDs	High Texture
1	0,12	0,15	0,12	4	1-4	
2	0,12	0,19	0,16	4	5-8	
3	0,15	0,3	0,2	4	9-12	
4	0,25	0,4	0,3	4	13-16	
5	0,3	0,5	0,4	4	17-20	
6	0,3	0,41	0,18	1	21	✓
7	0,12	0,375	0,15	4	22-25	✓
8	0,77	0,35	0,159	3	26-28	✓
9	0,41	0,652	0,244	1	29	✓
10	0,22	0,91	0,427	1	30	

**Fig. 12:**
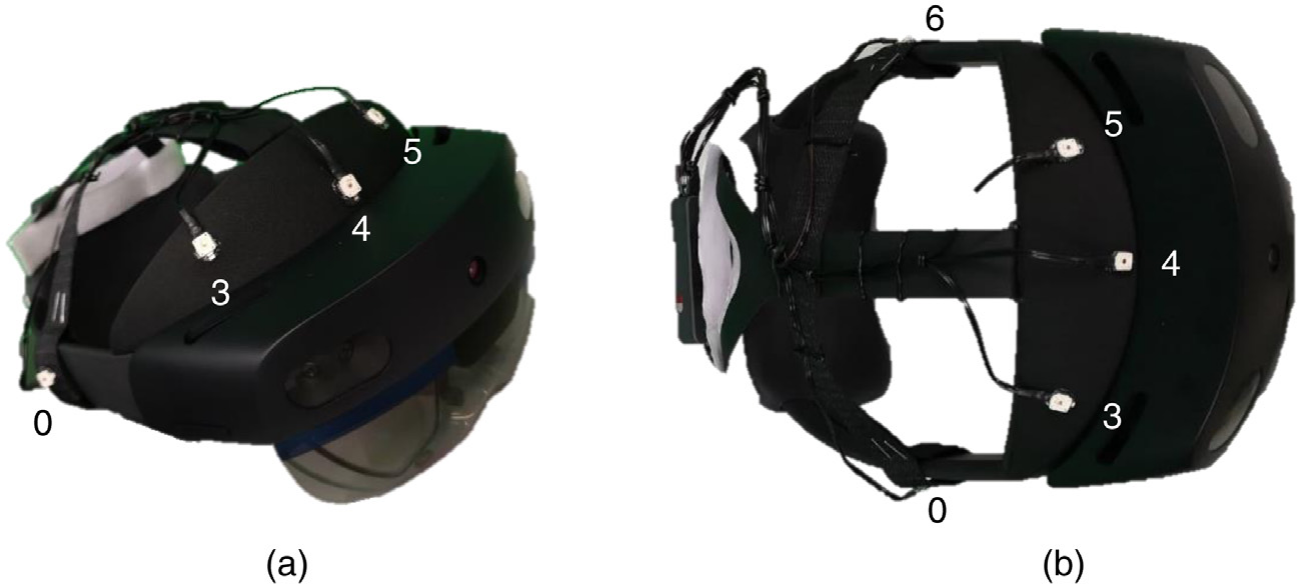
Identifiers and distribution of active markers on HL2 Device. (a) isometric view, (b) superior view.

**Cameras and acquisition systems**. Data was collected using Microsoft HoloLens 2, Kinect v2 device, and a motion capture system from phase space (MoCap). Specifications of those devices are presented in Table 3. A total of (2N+5) active markers were scanned during each session, where N represents the number of boxes in the session. For each box in the scene, two markers were attached to the most proximate superior corners, as depicted in Fig. 10. Five markers were placed on the HL2 device to track its pose, as illustrated in Fig. 12.

**Table 3:** Device specifications.

Device	Motion type	Cameras				
		#	FOV	Frequency	Resolution	Recording App
HoloLens 2 (HL2)	head mounted	5	DFOV 83°	Colour 30 Hz Depth [1-5] Hz	Gray Scale 640 × 480 Depth 512 × 512	StreamRecorder [8]
Kinect V2	fixed	2	Colour 84.1° × 53.8° Depth 70.6° × 60°	30 Hz	Color 1920 × 1980 Depth 512 × 424	Kinect SDK 2.0 [9]
PhaseSpace X2E (MoCap)	fixed	12	DFOV 60°	960 Hz	3600 × 3600	Master Client [10]

### 3.3 Methods

**Planning the consolidation zone**. The planning of the consolidation zone includes definitions of the type of boxes, number of instances and the pose of each box in the scene for each session. To solve this planning, an application was developed. This application takes as input: the desired level of factors for the session (see Table 1) and the available box types (see Table 2). Then, the application operates in three phases: (1) computes the IDs of the boxes that belong to the session, (2) randomizes the initial pose of the boxes in the consolidation zone, satisfying stacking and occlusion constraints and, (3) displays the results on a screen with a 3D animation.

**Manual Packing Operation.** A single male operator with a height of 1.65 m performed 28 manual packing sessions. Each session consists of identifying the target box in the consolidation zone, picking the target box, identifying the box's desired pose on the pallet, and transporting the box to the desired pose. The session ends when no more boxes are in the consolidation zone. At each step, the operator received visual instructions to perform the packing: the box ID to pick and the desired pose of the box on the pallet. Those instructions were computed with a commercial packing application, which returns the optimal packing pattern and the physical packing sequence (PPS). The instructions were displayed on screens placed near the working space.

**Raw Data Acquisition**. The procedure began with fixing the Kinect camera so that the consolidation zone was in the camera's field of view for all sessions. Then, an iterative procedure with seven steps was performed:

(1) Attach the markers to each box in the session. Two markers were attached to each box in the closest corners of the top face, as depicted in Fig. 10.
(2) Record the marker IDs in the MoCap recording application.
(3) Locate the boxes in predefined poses for the session at the consolidation zone. These poses are the output of the “planning the consolidation zone” procedure.
(4) Initiate recording applications on all devices.
(5) Perform manual packing until all the boxes are packed.
(6) Stop the recording applications on all devices
(7) Validate that the data were acquired successfully: if so, continue with the next session. Else, add the session number to the repetition list.

Finally, the sessions in the repetition list were re-collected with the seven iterative steps.

**Computing analyzed data from raw MoCap data (box 6D initial pose)**. An application was developed to compute the 6D pose of each box in a scene. This application uses as inputs the recorded 3D positions from MoCap markers and the descriptors of boxes that belong to the session (see Table 2). Initially, the application creates virtual boxes and locates each box assuming two constraints: (1) MoCap markers are always placed at the shortest edge on the top-most face of a box, and (2) the top-most faces of the boxes are always parallel to the ground plane. Note that four possible poses per box comply with those restrictions. The application allows the modification of the pose of each box by selecting from a predefined set of four possible poses. After the manual adjustment of poses (based on comparisons with captured color data from Kinect and HL2 devices), the application is used to export the pose of each box in the format illustrated in Fig. 5. When codifying the pose, two constraints were added to avoid pose-ambiguity: (1) the x-axis of each box is along the lower vertex of the top plane, (2) the angle between MoCap x-axis and box x-axis is in the range [-90 90].

**Computing filtered data from raw HL2 data (Point Clouds).** Point Clouds of some frames have been computed from raw HL2 data. Those frames correspond to frames that belong to the range init-frame and end-frame in the boxByFramet.txt file (see Fig. 6). The process was solved in three steps:

(1) Synchronization of pairs of color and depth images.
(2) Computation of colored point clouds with double precision from normalized raw images and intrinsic parameters.
(3) Transformation of point clouds to single and non-colored versions by using a standard library (Point Data Abstraction Library – PDAL [11]).

Steps 1 and 2 use algorithms available from the HL2 repository under the name StreamRecorder - Python postprocessing [8].

**Computing the projection matrix**$T_{h}^{m}$**.** The computation of the transformation matrix between the HL2 and the MoCap coordinate frame was solved in two phases: (1) gross approximation and (2) fine approximation. In the first phase the problem in equation (5) was formulated; this formulation required synchronization between samples of MoCap and HL2 devices,

(5) $$\begin{array}{c} {T_{h}^{m}}_{\mathrm{gross}}=T_{c_{h}}^{m}T_{h}^{c_{h}} \end{array}$$

$T_{c_{h}}^{m}$ is a rigid transformation that allows projecting a point from the HL2 camera rig coordinate frame ($q_{c_{h}}$) to MoCap coordinate frame ($q_{m}$). This transformation is approximated by processing the 3D positions associated with markers attached to the HL2 device (see Fig. 12). $T_{h}^{c_{h}}$ is a rigid transformation that allows projecting a point from the HL2 world coordinate frame ($q_{h}$) to the coordinate frame attached to the camera rig of the HL2 device ($q_{c_{h}}$). This transformation is computed by inverting the camera pose retrieved by the AR tracking algorithm available in the HL2 device [12]. In the second phase, the gross approximation was used as an initial value for an iterative closest point (ICP) procedure between (a) a synthetic point cloud referenced at MoCap coordinate frame ($q_{m}$), and (b) a scanned point cloud projected with $T{{}_{h}^{m}}_{gross}$. The output of this procedure is a transformation that allows us to refine the $T{{}_{h}^{m}}_{gross}$ result and obtain the $T_{h}^{m}$ shared in the dataset.

## Ethics Statements

This dataset does not contain information involving humans, animal experiments or data collection from social media platforms.

## CRediT authorship contribution statement

**Guillermo A. Camacho-Muñoz:** Conceptualization, Data curation, Methodology, Software, Investigation, Project administration, Writing – original draft. **Juan Camilo Martínez Franco:** Software, Visualization, Writing – review & editing. **Sandra Esperanza Nope-Rodríguez:** Conceptualization, Supervision, Methodology, Writing – review & editing. **Humberto Loaiza-Correa:** Conceptualization, Supervision, Methodology. **Sebastián Gil-Parga:** Software, Writing – review & editing. **David Álvarez-Martínez:** Conceptualization, Supervision, Writing – review & editing.

## Declaration of Competing Interest

The authors declare that they have no known competing financial interests or personal relationships that could have appeared to influence the work reported in this paper.

## Data Availability

6D-ViCuT: Six Degree-of-Freedom Visual Cuboid Tracking Dataset for Manual Packing of Cargo in Warehouses (Original data) (Dryad).
